# Glances at the Hospitals

**Published:** 1904-10-29

**Authors:** 


### GLANCES AT THE HOSPITALS.

### THE ROTHERHAM HOSPITAL.

The ordinary receipts amounted to £3,432, and the ordinary expenditure to £3,717, showing a deficiency of £285, which, added to the deficiency of the previous year made an adverse balance of £743. To reduce this the committee used £544 received as legacies, leaving a balance on the wrong side of £199. It is gratifying to be able to record that the workmen's own subscriptions show a considerable increase, and stand at £1,511 for the year. The average daily number resident was 45, and the average stay of each patient in the hospital was 25 days. We did not notice any statement of the cost per bed occupied nor of the cost per patient. The total number of in-patients was 653. The medical and surgical tables are fairly well arranged and a certain amount of information can be extracted, but it is not always so explicit as it might be. For instance we find "hernia and diseases of the alimentary system" lumped together in the operation table, and we are told there were 43 cases of these of whom 40 were relieved and 3 died. There were 14 amputations, 12 of these being returned as "relieved" and 2 died; but we are left in ignorance of the limbs or parts amputated. There was a total of 302 operations, and of this total 280 were relieved, 11 not relieved, and 11 died. There is no anæsthetic table.

### THE AYR COUNTY HOSPITAL.

The total income from every source amounted to £4,386, and the total expenditure to £4,217, showing that the income exceeded the expenditure by £169. This sum has been added to the Maintenance Reserve Fund which is now £754 The committee are to be congratulated on keeping their expenditure so well within their income, and they set an example too rarely followed in similar institutions. It is also deserving of notice that the workpeople's own contributions amounted to £316. The cost per bed occupied was £59 6s., the average cost per patient was £5 4s., and the average residence was 29 days. The average daily number resident was 68. At the beginning of the year 66 patients were in residence and 784 were admitted during the year, making a total of 850. Of these 622 were discharged cured or relieved, 24 not improved, 58 died, and 80 remained in the wards. Fever wards are attached to the infirmary; and we notice that of 28 cases of enteric 19 were cured, 6 died, and 3 remained under treatment. Of 27 cases of diphtheria 21 were cured, 3 died, and 3 remained. All the tables are carefully drawn out. 247 operations were performed. Of these 11 terminated fatally and 4 were moribund on admission. The operative mortality was 2·80 per cent. Chloroform was almost invariably used as the anæsthetic. On three occasions it was mixed with ether, and in one the A.C.E. mixture was employed.

### HOSPITAL FOR SICK CHILDREN, GREAT ORMOND STREET.

The ordinary income was £13,100, and the ordinary expenditure was £18,719, showing a deficit of £5,619. This deficit and the extraordinary expenditure of £2,990 was met by taking a surplus of £7,000 from the Imperial Bazaar account and by a balance in hand of £1,493. The committee say that the decrease in annual subscriptions is most disheartening. In spite of all their efforts they are unable to secure sufficient new annual subscriptions to meet the loss of others incurred every year. But we are glad to note that the donations and the legacies reached £4,892, a sum much higher than was received under this head during the previous year. The cost per bed occupied was £85 12s. 9d., which seems very high, although lower by £10 16s. than in 1902. The cost per patient was £5 13s., and the cost per week was £1 12s. 9d. The average duration of each patient's stay in the hospital was 24 days. It is satisfactory to see that the resources of the hospital were sufficient to meet the liabilities and that the present year began with a balance on the right side. Nevertheless the committee doubt whether they will be able to struggle through the year without an appeal for aid, and they close their report by saying "that in 1905 the sad necessity will once more become urgent of an appeal to the public for funds to carry on the hospital." We can only hope that such an appeal will not be necessary at an institution which does so much good, or if necessary that the appeal will be liberally responded to. The hospital contained at the beginning of the year 150 patients, and 2 403 were admitted during it. Of the total 1,055 were discharged cured, 825 were discharged relieved, 180 not relieved, 324 died, and 159 remained in the wards. A table of operations is given which shows the results in all cases. For appendicitis there were 21 operations. Of these 13 were cured, 2 relieved, 1 not relieved, and 5 died. Of 22 cases of laparotomy 12 were cured, 3 not relieved, and 7 died. The operation for the radical cure of hernia was performed 73 times, 67 being perfectly successful and 6 partially so. Altogether 945 operations were performed and there were 44 deaths. No anæsthetic table is given.

### TAUNTON AND SOMERSET HOSPITAL.

The general income was £5,068, and the general expenditure was £5,190, but there was a balance of £509 due to the treasurer at the close of the year. The New Building Fund account now reaches £7,573, but this will be insufficient to complete the work, and the committee appeal for further financial assistance under this head and doubtless this help will be forthcoming. At the beginning of the year there were 72 patients in the house, and 950 were admitted during it, making a total of 1,022. Of this number 714 were discharged cured or relieved, 24 not relieved, 22 for other reasons, 41 died, and 84 remained under treatment. The daily average number resident was 77, and the average stay in the hospital was 28 days. The cost per bed occupied is not given, nor the cost per patient. The demand for private nurses was greatly in excess of the supply; but the fees for services of those sent out amounted to £1,183. The medical and surgical statistics are contained on a single page and convey no useful information. It is much to be desired that this hospital should come into line with the best provincial hospitals and print tables which are useful for comparison and which enable the profession and the public to form some opinion of the value of the work done in the wards.

